# Non-linear self-driven spectral tuning of Extreme Ultraviolet Femtosecond Pulses in monoatomic materials

**DOI:** 10.1038/s41377-021-00531-8

**Published:** 2021-04-28

**Authors:** Carino Ferrante, Emiliano Principi, Andrea Marini, Giovanni Batignani, Giuseppe Fumero, Alessandra Virga, Laura Foglia, Riccardo Mincigrucci, Alberto Simoncig, Carlo Spezzani, Claudio Masciovecchio, Tullio Scopigno

**Affiliations:** 1 Graphene Labs, Istituto Italiano di Tecnologia, Via Morego 30, 16163 Genova, Italy; 2 Center for Life Nano Science @Sapienza, Istituto Italiano di Tecnologia, Viale Regina Elena 291, I-00161 Roma, Italy; 3 Dipartimento di Fisica, Università di Roma “La Sapienza”, Piazzale Aldo Moro 5, 00185 Roma, Italy; 4 Elettra-Sincrotrone Trieste S.C.p.A., SS 14-km 163.5, 34149 Basovizza Trieste, Italy; 5 Dipartimento di Scienze Fisiche e Chimiche, Università degli Studi dell’Aquila, Via Vetoio, 67100 L’Aquila, Italy

**Keywords:** X-rays, Nonlinear optics

## Abstract

Self-action nonlinearity is a key aspect – either as a foundational element or a detrimental factor – of several optical spectroscopies and photonic devices. Supercontinuum generation, wavelength converters, and chirped pulse amplification are just a few examples. The recent advent of Free Electron Lasers (FEL) fostered building on nonlinearity to propose new concepts and extend optical wavelengths paradigms for extreme ultraviolet (EUV) and X-ray regimes. No evidence for intrapulse dynamics, however, has been reported at such short wavelengths, where the light-matter interactions are ruled by the sharp absorption edges of core electrons. Here, we provide experimental evidence for self-phase modulation of femtosecond FEL pulses, which we exploit for fine self-driven spectral tunability by interaction with sub-micrometric foils of selected monoatomic materials. Moving the pulse wavelength across the absorption edge, the spectral profile changes from a non-linear spectral blue-shift to a red-shifted broadening. These findings are rationalized accounting for ultrafast ionization and delayed thermal response of highly excited electrons above and below threshold, respectively.

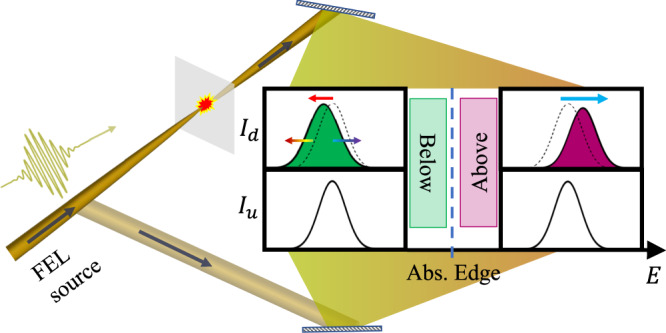

## Introduction

Progresses in non-linear optics have been facilitated by the development of high peak power, table-top pulsed laser sources operating in a relatively narrow spectral range spanning from the infrared to the ultraviolet. Such spectral constraint for fs-lasers restricts the interaction processes with condensed matter to those involving barely bound valence band electrons, limiting, in turn, the development of non-linear spectroscopies and photonic devices.

A variety of non-linear interaction mechanisms occurs between light and matter in presence of intense radiation fields^1^, which may alter the transient optical properties experienced by the light propagating in the material. The most elemental non-linear effect (NLE), arising at the single pulse level, is the self-induced spectral modulation, which produces the modification of the pulse spectral properties due to the polarizability induced in the matter by the pulse itself. Spectral modulation can originate from different optical phenomena, including Raman induced self-frequency shift, optical shock formation, and self-phase modulation (SPM)^1^. This latter, in particular, represents one of the primary tools used for tuning the spectral bandwidth at visible wavelengths by Kerr effect in transparent media. Briefly, the presence of a strong laser field induces an intensity-dependent modulation of the material’s refractive index which, in turn, produces a non-linear phase shift on the electromagnetic field, resulting in a broadening of its spectral profile^1^. Used in combination with chirped mirrors, for example, SPM allows producing high intensity ultrashort optical pulses. On the other hand, it is detrimental in chirped pulse amplification^2^, where it significantly distorts the recompressed pulse, limiting peak power, and pulse contrast. Kerr effect also sets the temporal resolution limit in time-resolved spectroscopies, due to pump-probe cross phase modulation^3,4^.

Exploring novel non-linear light-matter interactions in EUV and X-ray ranges, enabled by FEL and HHG sources, is recently attracting a lot of interest, based on the opportunity to design new spectroscopic approaches^5–7^ and to exploit NLEs in a high-energy regime^8–16^. For example, theoretical and experimental efforts have been devoted to probe the effects of the core dynamics on the absorption properties of the sample, monitoring the photo-induced modification to the absorption spectra of materials pumped in the XUV and EUV ranges, either by the strong field of a single probe pulse^8,12,17^ or by using an additional pump pulse^18^. Differently, evidences for plasma induced SPM under *valence* electrons photo-ionization in gas phase^19^ or free carrier generation in transparent materials^20,21^ have been reported under visible excitation only: creation of plasmas by intense laser pulses and consequent laser-plasma interactions involve highly non-linear processes, producing a spectral blue-shift. Critically, the lack of EUV and X-ray femtosecond pulses with controlled spectral and temporal profiles has prevented the observation of the self-induced non-linear interactions with core electrons in non transparent materials.

Here, we demonstrate self-induced intrapulse dynamics of EUV seeded-FEL pulses propagating in sub-micrometer self-standing sample foils. Taking advantage of high peak power, transversal and longitudinal coherence and spectral stability of seeded-FEL sources^22^, we exploit the NLEs in materials with metallic properties to alter the spectral shape of the transmitted FEL pulse. Interestingly, this phenomenon reveals a strong dependence on the interaction process between light and core electrons. Specifically, we observed an asymmetric red-shifted spectral broadening when exposing the sample to an intense EUV pulse with photon energy below a selected core absorption edge. This effect is rationalized by modeling SPM and delayed thermal response of electrons (DTRE)^23^, enabling the measurement of non-linear parameters of the sample material. On the other hand, increasing the EUV photon energy a few eV above a targeted absorption edge, we measured a pronounced non-linear blue-shift, assigned to a SPM effect induced by photo-induced core electron ionization.

**Fig. 1:**
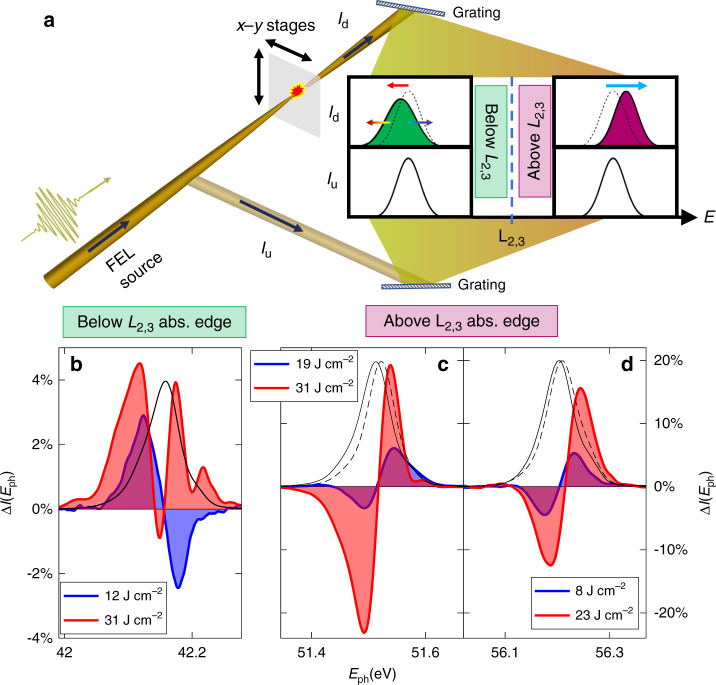
Experimental results in Mg. **a** Simplified sketch of the experimental setup (see Methods section). The single-shot spectra of the pulses are collected with two spectrometers (depicted as a couple of gratings) upstream (*I*_u_(*E*_ph_)) and downstream (*I*_d_(*E*_ph_)) of a Mg sample foil. The NLEs are obtained comparing the two FEL spectra, demonstrating a spectral blue-shift above the *L*_2,3_ absorption edge. On the contrary, spectral red-shift and broadening can be observed below edge. The averages of the differences Δ*I* = 〈*I*_d_ − *I*_u_〉 is reported for a FEL photon energy (~42.1 eV) below the Mg L_2,3_-edge (**b**) and for two photon energies (~51.5 and ~56.2 eV) above it (**c**, **d**). The red and blue curves correspond to Δ*I* at the highest and lowest fluence. Averaged FEL spectra 〈*I*_u_(*E*_ph_)〉, scaled to improve readability, are shown by solid black lines. The blue-shift in c,d can also be appreciated comparing 〈*I*_u_(*E*_ph_)〉 and 〈*I*_d_(*E*_ph_)〉 at the largest fluence (dashed black lines).

The experiment, sketched in Fig. 1a, is carried out at the beamline EIS-TIMEX of the FERMI FEL in Trieste (Italy)^24^. FERMI delivers intense nearly transform-limited sub-100-fs pulses, finely tunable in wavelength and intensity (see Methods section for details). The pulses are focused on free-standing sub-micrometric (100–300 nm) sample foils of metals and semiconductors, mounted on supporting rings. Particularly, measurements at different photon energies in the range 32–56 eV and different fluences are performed on Mg and Se that exhibit sharp absorption edges (*L*_2,3_ ~ 50 eV and *M*_4,5_ ~ 55 eV, respectively^25^) in the experimental spectral window. Every single FEL pulse is monitored by two spectrometers. The first one, located upstream of the experimental chamber, measures the FEL spectral intensity (*I*_u_(*E*_ph_)) before the interaction with the sample. The second one, placed downstream of the chamber, performs an analogous measurement for the light transmitted by the sample (*I*_d_(*E*_ph_)). In front of this latter spectrometer a rotating wheel with different metallic filters preserves the light sensors from saturation. The averages of differential single-shot normalized spectra (Δ*I*(*E*_ph_) = 〈*I*_d_(*E*_ph_) − *I*_u_(*E*_ph_)〉, see Methods section) for a 140-nm-thick Mg self-standing foil, protected on both sides by an Al coating (19 nm)^12^, is reported in Fig. 1b–d.

**Fig. 2:**
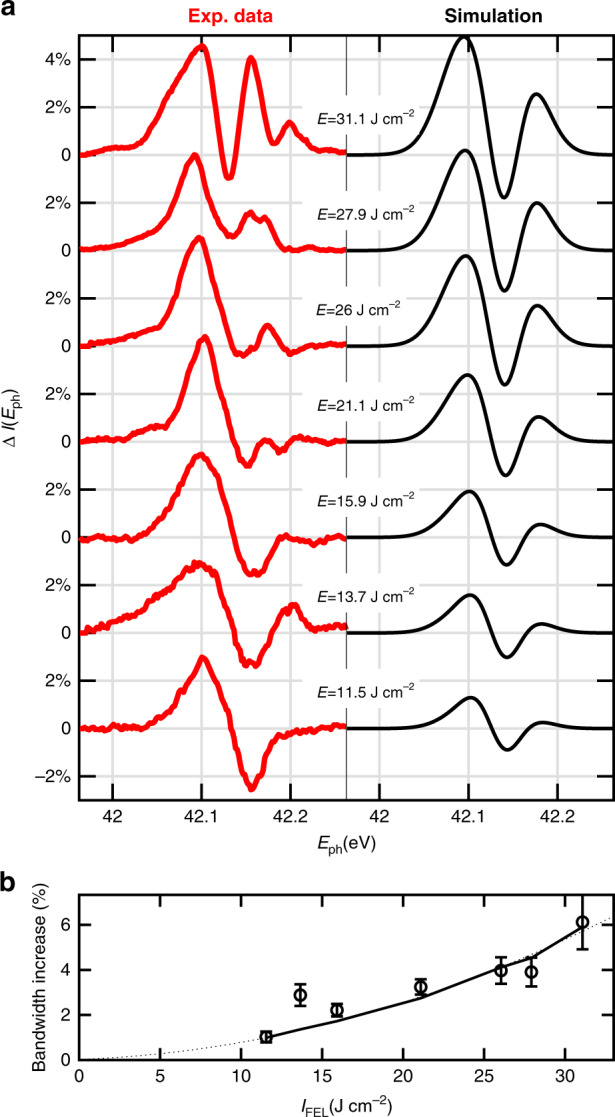
Theoretical simulation of experimental results below the absorption edge. **a** The experimental data (red lines) reported for different FEL fluences in 140 nm of Mg are compared with the relative simulation (black lines) of DTRE and SPM NLEs obtained from a fitting procedure of Eq. (6). The modification of pulse intensity (Δ*I* = ∣*ψ*(*L*, *E*_ph_)∣^2^ − ∣*ψ*(0, *E*_ph_)∣^2^) is calculated (see Methods section). **b** The percentage increases of bandwidth, observed in our measurements (circles) and in the theoretical curves (solid line), are depicted. The dotted line is a parabolic guide to the eye.

Notably, the Δ*I* signal in Mg reveals a different system response upon light-excitation above and below *L*_2,3_ absorption edge. As shown by the raw spectra in Fig. 1c, d (〈*I*_u_(*E*_ph_)〉, solid black line, and 〈*I*_d_(*E*_ph_)〉, dashed black line), a blue-shift dominates the non-linear spectral modification above the edge. Accordingly, the Δ*I*(*E*_*ph*_) profiles (colored blue and red areas) show dispersive lineshapes. This is opposed to the case of gas photo-ionization in the visible, where there exists an initial propagation regime in which plasma generation is negligible and Kerr SPM dominates^19^. Below the *L*_2,3_-edge, the spectral shape of Δ*I*(*E*_ph_) is more complex and fluence dependent, as reported in Fig. 1b. Specifically, at the highest FEL fluence, Δ*I*(*E*_ph_) exhibits two positive lobes, signature of a spectral broadening (quantified in Fig. 2b). Upon decreasing the FEL fluence, Δ*I*(*E*_ph_) gradually evolves to a dispersive shape, corresponding to a spectral red-shift.

We ascribe the observed dependence of the spectral behavior on the photon frequency to the presence of a core absorption edge in the explored spectral region, drastically altering the interaction of EUV photons with the material. In order to rationalize the measured spectral shifts, we model the data by describing optical pulse propagation in the non-linear medium. When an optical field ${\bf{E}}({\bf{r}},t)={\rm{Re}}[\psi ({\bf{r}},t){{\rm{e}}}^{i{k}_{0}z-i{\omega }_{0}t}\hat{{\bf{n}}}]$ with generic complex polarization unit vector $\hat{{\bf{n}}}$, optical envelope *ψ*(**r**, *t*), carrier angular frequency *ω*_0_, and wave-vector *k*_0_ = *ω*_0_*n*(*ω*_0_)/*c* propagates in a non-linear medium with linear refractive index *n*(*ω*_0_), it undergoes a self-induced phase shift^1^ quantified as

1 $${\phi }_{{\rm{NL}}}({\bf{r}},t)={\chi }^{(3)}| \psi ({\bf{r}},t){| }^{2}{k}_{0}L$$

where *L* is the sample thickness, and *χ*^(3)^ is the third-order Kerr susceptibility, which represents the light capability to modify the refractive index of the absorbing material. This time-dependent non-linear phase shift, induced by a non-linear polarization (**P**_NL_(**r**, *t*)), leads to self-induced spectral broadening of ultrashort pulses^26^. As shown in Eq. (1), Kerr-induced SPM is proportional to the *χ*^(3)^ of the material. Owing to resonant interband electron dynamics, in the visible range metals exhibit higher *χ*^(3)^ values (~10^−16^ m^2^V^−2^) than semiconductors (2.8 × 10^−18^ m^2^V^−2^ in silicon), glasses (~10^−22^ m^2^V^−2^) and solvents (~10^−20^ m^2^V^−2^)^27^. Such highly resonant non-linear behavior in metals cannot be efficiently exploited in the visible range, as it is accompanied by poor light transmission with the noteworthy exception of FWM in graphene^28–30^. This forces resorting to surface plasmon polariton waves to obtain non-linear functionalities in metal-based table-top photonic systems^31^. EUV and X-ray photons are instead well transmitted by sub-micrometric metallic media and can excite core photoelectrons, potentially representing the most suitable platform for full exploitation of NLEs in metals.

In our experiment, when the EUV FEL photon energy slightly exceeds selected core electron binding energies (above-edge condition), core photoelectrons are massively promoted nearly above the Fermi level, possibly leading to saturable absorption^12^. In this condition, photoelectrons form a transient hot dense ionized plasma, which gives rise to a SPM different than that originated by Kerr effects in transparent media. In the case of free electron plasma generation driven by the pulse absorption, the charges induce a sudden decrease of the refractive index in the system^32,33^. Such an absorption-driven effect induces a non-linear modification of the light propagation, accelerating the trailing edge of the pulse and consequently blue-shifting the spectrum. Specifically, the leading edge of the pulse propagates through an absorbing medium, while the trailing part, due to the free-electron dispersion produced by the leading front, experiences a reduced refractive index and is hence accelerated. As a result of such asymmetric temporal compression the pulse spectral components are blue-shifted, which rationalizes the experimental results in Fig. 1c, d.

Below the absorption edge, the sample absorption coefficient for EUV photons is radically lower and the electron plasma density is negligible, leading to the suppression of the intense spectral blue-shift. Nevertheless, spectral NLEs, induced by the high EUV local electric field in the excited sample volume, can still be observed. Specifically, a clear spectral broadening (red curves in Fig. 1b) is obtained at high FEL fluence. Such an effect can be rationalized in terms of Kerr-induced SPM under non absorbing conditions. However, SPM alone cannot account for the spectral red-shift occurring at low fluence, as demonstrated by simulations in Fig. S1. Under low local electric field excitation, spectral red-shifts can be explained in terms of DTRE^23,34^. DTRE is frequently observed in plasmas, doped semiconductors, and metals, because optical interaction with electrons leads to ultrafast out-of-equilibrium electronic heating, which modulates the refractive index with a delayed response, arising from the excitation of hot conduction electrons (*τ*_th_ = 1 fs) and its consequent relaxation to equilibrium via electron-phonon scattering (*τ*_r_ = 100 fs)^35,36^. Owing to DTRE, the temporal relaxation dynamics is accompanied by a non-linear spectral red-shift in the frequency domain^23^.

Such a complex ultrafast non-linear dynamics, triggered by the concomitant SPM and DTRE effects, can be phenomenologically modeled through a two-temperature model^23,34^, which determines the following time-dependent non-linear polarization (see Methods section):

2 $${{\bf{P}}}_{{\rm{NL}}}({\bf{r}},t)={\epsilon }_{0}{\rm{Re}}\left\{{\chi}^{(3)}\left[(1-{f}_{\rm{T}})| \psi ({\bf{r}},t)|^{2} + \, {f}_{{\rm{T}}}\displaystyle{\int\nolimits_{0}^{\infty}}dt^{\prime} {h}_{{\rm{T}}}(t^{\prime})| \psi ({\bf{r}},t-t^{\prime} )|^{2}\right]\psi ({\bf{r}},t){{\rm{e}}}^{i{k}_{0}z-i{\omega }_{0}t}\hat{{\bf{n}}}\right\}$$

where *f*_T_ is the thermal fraction and ${h}_{{\rm{T}}}(t)={({\tau }_{{\rm{th}}}-{\tau }_{{\rm{r}}})}^{-1}\left({e}^{-t/{\tau }_{{\rm{th}}}}-{e}^{-t/{\tau }_{{\rm{r}}}}\right)$ is the thermal response function. In Fig. 2a we report the spectral modifications induced by sample nonlinearity, for selected fluence values. Experimental data are best represented by simulations obtained propagating a 59-fs transform-limited pulse in a material with *f*_T_ = 0.29, and *χ*^(3)^ = 1.45 × 10^−23^ m^2^V^−2^ and carrier photon energy of 42.12 eV.

Notably, such *χ*^(3)^ value is larger than the one measured in Si_3_N_4_ by transient FWM^13^ (6 × 10^−24^ m^2^V^−2^), indicating stronger NLE in metals. Moreover, the DTRE effect, related to the interaction with free electrons, has a tangible role below the absorption edges, as expected for metals in the visible range. The extra oscillations observed in the experimental data at ~42.2 eV can be ascribed to spectral deviation of the FEL source with respect to an ideal transform-limited Gaussian pulse, as discussed in supplementary information. As shown in Fig. 2, the role of DTRE contribution is more evident in the energy region up to 16 J cm^−2^. The measured *f*_T_ implies that thermal excitation of electrons is comparable in efficiency to the instantaneous Kerr nonlinearity. In addition, our results indicate that hot electron thermalization occurs in the fs timescale and consequently collision-induced dephasing plays a major role in the electron dynamics, thus preventing coherent effects like Rabi oscillations or self-induced transparency^37^.

**Fig. 3:**
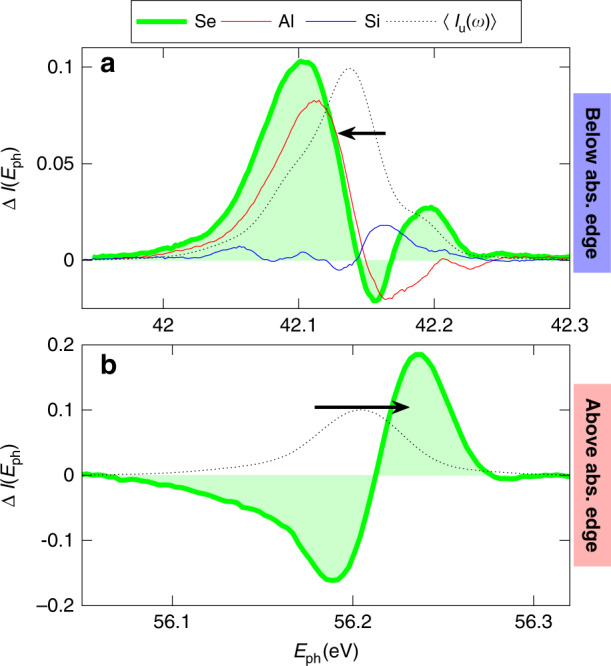
Spectral modification in sub-micrometric films. **a** The difference of normalized spectra before and after the sample interaction in a 300 nm Al film (red line), 100 nm of Si (blue line), and 120 nm of Se (green area) at 42.1 eV with a fluence of 31 J cm^−2^. **b** The measurement is performed in Se film above the M_4,5_ absorption edge (at 55 eV) with a fluence of 9 J cm^−2^. 〈*I*_u_(*E*_ph_)〉 of the sample, divided by a factor 10, is depicted by the black dotted lines. The horizontal black arrows indicate the shift direction.

Similar measurements are carried out at ~42.1 eV for other sub-micrometric sample foils, in order to extend the experimental investigation to a wider class of materials. In particular, in Fig. 3a we report Δ*I* measured in Al (300 nm), Si (100 nm), and Se (120 nm). In agreement with the Mg case, since the experimental photon energy is below the core binding energy of the three materials, all the measurements do not show any spectral blue-shift. Al and Se exhibit a spectral modification, while no substantial effect is observed in Si, confirming the higher *χ*^(3)^ of metallic samples also in EUV regime, as previously suggested by the comparison with transient FWM experiment^13^. Furthermore, measurements in Se above the M_4,5_ absorption edge (see Fig. 3b) provide an additional evidence of plasma induced spectral blue-shift. Further improvement of the NLEs efficiency can be achieved using thicker samples and photo-exciting below the absorption edge. In particular, the extracted Mg *χ*^(3)^ indicates that a micrometer sample can generate an increase of the FEL full width half maximum up to a 2.5 factor (0.14 eV).

In summary, we have explored the sub-picosecond non-linear response of Mg and Se in the extreme ultraviolet and, focusing high fluence EUV beams into opaque sub-micrometric foils, we have shown how to exploit NLEs for driving a self-induced modification of the pulse spectrum. Specifically, we reveal a plasma induced blue-shift when the FEL pulse photon energy exceeds core electrons binding energy. In striking contrast, when core electron photo-absorption is not activated, two different regimes are observed: a red-shift at low fluences (<16 J cm^−2^), and a spectral broadening at higher fluences. This intensity dependence is rationalized accounting for the concurring presence of two different non-linear processes, namely Kerr-induced SPM and DTRE, allowing to measure the thermal fraction, ~30% in Mg, and the third-order nonlinearity, ${\chi }_{{\rm{Mg}}}^{(3)}=1.45\times 1{0}^{-23}$ m^2^V^−2^. The presented results, verified in different materials, provide the first evidence of self-induced spectral beam modification in the EUV, indicating the crucial role of the core electron absorption edges for such NLEs and demonstrating the higher non-linear efficiency in metallic samples, compared to semiconducting materials. We anticipate that the full exploitation of these NLEs will lead to a fine control of the EUV ultrashort light pulse spectral profile in FEL facilities or in table-top HHG setups, creating new opportunities in non-linear photonics and time-resolved spectroscopies^5,7,38^.

## Methods

### Experimental setup

The FERMI FEL source is optimized to deliver nearly transform-limited EUV pulses with an intensity of ~75 μJ, measured by a calibrated N_2_ ionization chamber. The system is able to emit photons from 12 eV to 62 eV, with the pulse duration that depends on the photon energy. In our experiment, they are 42.1, 51.5, and 56.2 eV and 59, 55, and 54 fs, respectively^22^. The FEL beam is delivered to the end-station using grazing incidence (~2°) carbon coated mirrors. The experimental setup consists of two EUV single-shot spectrometers positioned upstream (PRESTO) and downstream (WEST) of an experimental chamber where the sample is accommodated. The resolving power of the two spectrometers is ~10^4^, appropriate to monitor the FEL peak profiles with typical bandwidth of ~0.03 nm. No monochromators are involved in the setup. The FEL beam is focused on the sample by a gold coated ellipsoidal mirror with a focal length of 1400 mm. This tight focusing in a region of diameter ~11μm permits reaching fluences of about ~31 J cm^−2^. Absorption of intense FEL pulses induces a permanent damage^12^ on samples, therefore measurements have been carried out in single shot fashion. In the probed spectral region, the above threshold absorption is practically constant (<2%). Therefore, it does not contribute to spectral distortions, which can only be ascribed to non-linear effects. After each FEL shot, selected by a mechanical fast shutter, the sample is moved to an unexposed region, using an automatically moving 5-axes manipulator preserving both the focus and the normal incidence of the FEL beam. Single FEL shots are selected with a repetition rate of ~0.25 Hz by a mechanical fast shutter with a diamater of 6 mm positioned between the sample and the ellipsoidal mirror. The normalized spectra before *I*_u_(*E*_ph_) and after *I*_d_(*E*_ph_) the sample are collected for each single FEL shot. Preliminary, *I*_u_(*E*_ph_) and *I*_d_(*E*_ph_) are both energy shifted to maximize the spectral overlap of different shots at the upstream detector, which is then used as a reference: in fact, self-induced spectral modifications are obtained averaging single-shot spectral differences 〈*I*_d_(*E*_ph_) − *I*_u_(*E*_ph_)〉 over ~100 FEL shots.

To mitigate artifacts from the long-term fluctuations of the spectrometers’ calibration, we collected short sequences (1–2 min) of calibration and measurement data subsequently. The averaged difference between the simultaneous normalized measurements of the two spectrometers, reported in Figs. 1 and 3, have been corrected by subtracting the calibration spectrum.

### Theoretical calculations

For photon energies below the ionization threshold, in order to account for the ultrafast dynamics of Mg, we adopt a phenomenological two-temperature model^23,34^

3 $$\dot{{\mathcal{N}}}=-{\tau }_{{\rm{th}}}^{-1}{\mathcal{N}}+{P}_{{\rm{A}}}$$

4 $${\dot{T}}_{{\rm{e}}}={\tau }_{{\rm{r}}}^{-1}({T}_{{\rm{eq}}}-{T}_{{\rm{e}}})+({\gamma }_{{\rm{e}}}/{C}_{{\rm{e}}}){\mathcal{N}}$$

describing the temporal evolution of non-thermalized electrons with energy density ${\mathcal{N}}({\bf{r}},t)$ thermalizing at rate ${\tau }_{{\rm{th}}}^{-1}$ to the out-of-equilibrium temperature *T*_e_(**r**, *t*) and subsequent relaxation to the equilibrium lattice temperature *T*_eq_ at rate ${\tau }_{{\rm{r}}}^{-1}$ upon radiative excitation by an electromagnetic pulse with electric field ${\bf{E}}({\bf{r}},t)={\rm{Re}}[\psi ({\bf{r}},t){{\rm{e}}}^{i{k}_{0}z-i{\omega }_{0}t}\hat{{\bf{n}}}]$, where *ψ*(**r**, *t*) is the optical envelope, *ω*_0_ is the carrier angular frequency, *k*_0_ = *ω*_0_/*c* is the carrier wavevector, and $\hat{{\bf{n}}}$ is the polarization unit vector. The absorbed power per unit volume averaged over fast temporal oscillations is *P*_A_(**r**, *t*) = (1/2)*ϵ*_0_*ϵ″*(*ω*_0_)*ω*_0_∣*ψ*∣^2^, where *ϵ*(*ω*_0_) is the metal linear dielectric constant at the carrier angular frequency and the double prime indicates the imaginary part, while *γ*_e_ represents the electron-electron collision rate and *C*_e_ is the electron heat capacity per unit volume. Considering the previous equation, the absorbed power *P*_A_(**r**, *t*) depends on time over the timescale of the radiation envelope (59 fs). The two-temperature model is derived from first-principles by the method of moments starting directly from the Boltzmann equations for lattice and free electron fluids in the relaxation approximation (non-ideal plasma, electron-electron and electron-phonon collisions are phenomenologically accounted for by the relaxation rates)^23^. Such model can be analytically solved for arbitrary pulses by Fourier transform, leading to the temporal evolution of the electron temperature variation

5 $${{\Delta }}{T}_{{\rm{e}}}(t)={T}_{{\rm{e}}}(t)-{T}_{{\rm{eq}}}={C}_{{\rm{e}}}^{-1}{\gamma }_{{\rm{e}}}{\tau }_{{\rm{r}}}{\tau }_{{\rm{th}}}{\int\nolimits_{0}^{\infty }}dt^{\prime} {h}_{{\rm{T}}}(t^{\prime} ){P}_{{\rm{A}}}(t-t^{\prime} )$$

where ${h}_{{\rm{T}}}(t)={({\tau }_{{\rm{r}}}-{\tau }_{{\rm{th}}})}^{-1}\left({e}^{-t/{\tau }_{{\rm{r}}}}-{e}^{-t/{\tau }_{{\rm{th}}}}\right)$ is the thermal response function, *τ*_th_ (of the order of fs) is the electron thermalization time arising from collisions that at the same time produce electronic dephasing. After ultrafast heating, the increased electron temperature *T*_e_ relaxes to the lattice temperature with the characteristic time *τ*_r_, accounting for electron-phonon collisions. The main limitation of the model lies in its inadequacy to produce meaningful predictions for radiation pulses with time duration shorter than *τ*_th_ because in such regime the electron distribution is utterly non-thermal and the definition of an out-of-equilibrium hot electron temperature is meaningless. However, in our present investigation, the radiation pulse duration is longer than *τ*_th_ and hence the model is fully adequate to produce accurate predictions. Further, the model is spatially local, assumes a homogeneous lattice, and neglects hot-electrons diffusion occurring over timescales longer than our pulse duration^39,40^.

The ultrafast electron heating introduces a non-linear modulation of the metal dielectric constant, provided at first order by *δϵ*(**r**, *t*) = *κ*Δ*T*_e_(**r**, *t*), where *κ* is the complex thermo-derivative coefficient^23,34^. In turn, accounting also for Kerr nonlinearity arising from the instantaneous conduction hot-electron response, one gets the non-linear polarization **P**_NL_(**r**, *t*) (reported in Eq. (2)), in which the coefficients *χ*^(3)^*f*_T_ contain all the time independent terms of the two-temperature model.

We calculate radiation propagation in the medium through the Generalized Nonlinear Schrödinger Equation (GNLSE) for the envelope *ψ*(**r**, *t*). Starting from Maxwell’s equations coupled with conduction hot-electron dynamics through the non-linear polarization **P**_NL_(**r**, *t*), radiation evolution is described by the inhomogeneous D’Alambert equation ∇ × ∇ × **E** = − *μ*_0_∂^2^**P**_NL_/∂*t*^2^ − (*ϵ*(*ω*_0_)/*c*^2^)∂^2^**E**/∂*t*^2^. Thus, adopting the slowly varying envelope approximation (SVEA), one gets to the following evolution equation for the envelope

6 $${\partial }_{z}\psi (z,t)=i\frac{{k}_{0}}{2}{\chi }^{(3)}\left[(1-{f}_{{\rm{T}}})| \psi (z,t)|^{2} + \,{f}_{{\rm{T}}}{\displaystyle\int\nolimits_{0}^{\infty }}dt^{\prime} {h}_{{\rm{T}}}(t^{\prime} )| \psi (z,t-t^{\prime} )|^{2}\right]\psi (z,t)-\frac{A}{2}\psi (z,t)$$

where *A* is the Mg attenuation coefficient. Diffraction and dispersion are neglected owing to the nanometer scale propagation that prevents such effects to play any role. Furthermore, we have assumed that the linear refractive index is *n*(*ω*_0_) ≃ 1, which is consistent with transmission measurements of Mg at the frequency range of interest. The theoretical results reported above are obtained through a numerical solution of Eq. (6), implemented by a fourth-order Runge-Kutta algorithm complemented with fast Fourier transform. We assume for *ψ*(0, *t*) a Gaussian transform-limited profile with the experimental time duration (59 fs). The Fourier transform *ψ*(0, *E*_ph_) is compatible with the average experimental spectral profile (see supplementary information). The experimental data are compared with the difference of normalized ∣*ψ*(*L*, *E*_ph_)∣^2^ and ∣*ψ*(0, *E*_ph_)∣^2^ (Δ∣*ψ*(*L*, *E*_ph_)∣^2^).

## Supplementary information

Supplementary information

## References

[1] Agrawal, G. *Nonlinear Fiber Optics. 4th edn*. (Academic Press, 2012).

[2] Strickland D, Mourou G (1985). Compression of amplified chirped optical pulses. Opt. Commun..

[3] Ekvall K (2000). Cross phase modulation artifact in liquid phase transient absorption spectroscopy. J. Appl. Phys..

[4] Batignani G (2019). Genuine dynamics vs cross phase modulation artifacts in femtosecond stimulated raman spectroscopy. ACS Photon..

[5] Kowalewski M (2017). Simulating coherent multidimensional spectroscopy of nonadiabatic molecular processes: from the infrared to the x-ray regime. Chem. Rev..

[6] Mukamel S, Healion D, Zhang Y, Biggs JD (2013). Multidimensional attosecond resonant x-ray spectroscopy of molecules: Lessons from the optical regime. Annu. Rev. Phys. Chem..

[7] Rohringer N (2019). X-ray raman scattering: a building block for nonlinear spectroscopy. Proc. R. Soc. A Math. Phys. Eng. Sci..

[8] Nagler B (2009). Turning solid aluminium transparent by intense soft X-ray photoionization. Nat. Phys..

[9] Glover T (2012). X-ray and optical wave mixing. Nature.

[10] Weninger C (2013). Stimulated electronic x-ray raman scattering. Phys. Rev. Lett..

[11] Shwartz S (2014). X-ray second harmonic generation. Phys. Rev. Lett..

[12] Mincigrucci R (2015). Role of the ionization potential in nonequilibrium metals driven to absorption saturation. Phys. Rev. E.

[13] Foglia L (2018). First evidence of purely extreme-ultraviolet four-wave mixing. Phys. Rev. Lett..

[14] Lam RK (2018). Soft x-ray second harmonic generation as an interfacial probe. Phys. Rev. Lett..

[15] Bencivenga F (2019). Nanoscale transient gratings excited and probed by extreme ultraviolet femtosecond pulses. Sci. Adv..

[16] Fidler AP (2019). Nonlinear XUV signal generation probed by transient grating spectroscopy with attosecond pulses. Nat. Commun..

[17] Ott C (2019). Strong-field extreme-ultraviolet dressing of atomic double excitation. Phys. Rev. Lett..

[18] Ding T (2019). Nonlinear coherence effects in transient-absorption ion spectroscopy with stochastic extreme-ultraviolet free-electron laser pulses. Phys. Rev. Lett..

[19] Hölzer P (2011). Femtosecond nonlinear fiber optics in the ionization regime. Phys. Rev. Lett..

[20] Roy S, Marini A, Biancalana F (2013). Self-frequency blueshift of dissipative solitons in silicon-based waveguides. Phys. Rev. A.

[21] Blanco-Redondo A (2014). Controlling free-carrier temporal effects in silicon by dispersion engineering. Optica.

[22] Finetti P (2017). Pulse duration of seeded free-electron lasers. Phys. Rev. X.

[23] Marini A (2013). Ultrafast nonlinear dynamics of surface plasmon polaritons in gold nanowires due to the intrinsic nonlinearity of metals. New J. Phys..

[24] Masciovecchio C (2015). EIS: the scattering beamline at FERMI. J. Synchrotron Radiat..

[25] Thompson, A. C. & Vaughan, D. *X-ray Data Booklet* (Lawrence Berkeley National Laboratory, University of California Berkeley, 2001).

[26] Alfano RR, Shapiro SL (1970). Observation of self-phase modulation and small-scale filaments in crystals and glasses. Phys. Rev. Lett..

[27] Boyd, R. W. *Nonlinear optics*, 3th edn. (Academic press, 2018).

[28] Hendry E (2010). Coherent nonlinear optical response of graphene. Phys. Rev. Lett..

[29] Lafetá L (2017). Anomalous nonlinear optical response of graphene near phonon resonances. Nano Lett..

[30] Virga A (2019). Coherent anti-Stokes Raman spectroscopy of single and multi-layer graphene. Nat. Commun..

[31] Kauranen M, Zayats AV (2012). Nonlinear plasmonics. Nat. Photon..

[32] Penetrante BM (1992). Ionization-induced frequency shifts in intense femtosecond laser pulses. J. Opt. Soc. Am. B.

[33] Baudisch M (2018). Ultrafast nonlinear optical response of dirac fermions in graphene. Nat. Commun..

[34] Marini A, Ciattoni A, Conti C (2019). Out-of-equilibrium electron dynamics of silver driven by ultrafast electromagnetic fields - a novel hydrodynamical approach. Faraday Discuss..

[35] Cai W, Shalaev V (2010). Optical Metamaterials: Fundamentals and Applications..

[36] Mueller BY, Rethfeld B (2013). Relaxation dynamics in laser-excited metals under nonequilibrium conditions. Phys. Rev. B.

[37] Marini A, Biancalana F (2013). Ultrashort self-induced transparency plasmon solitons. Phys. Rev. Lett..

[38] Bressler C, Chergui M (2004). Ultrafast x-ray absorption spectroscopy. Chem. Rev..

[39] Block A (2019). Tracking ultrafast hot-electron diffusion in space and time by ultrafast thermomodulation microscopy. Sci. Adv..

[40] Najafi E, Ivanov V, Zewail A, Bernardi M (2017). Super-diffusion of excited carriers in semiconductors. Nat. Commun..

