# Supplementary material for: Non-linear self-driven spectral tuning of Extreme Ultraviolet Femtosecond Pulses in monoatomic materials
Source: Light Sci Appl. 2021 Apr 28;10:92. doi: 10.1038/s41377-021-00531-8 (PMC8080687; doi:10.1038/s41377-021-00531-8)
Supplement: Supplementary file 1 — Supplementary information [file 41377_2021_531_MOESM1_ESM.pdf]

Non-linear self-driven spectral tuning of Extreme Ultraviolet Femtosecond Pulses in monoatomic materials - Supplementary information

Carino Ferrante[1,2,3*], Emiliano Principi[4], Andrea Marini[5], Giovanni Batignani[3], Giuseppe Fumero[3], Alessandra Virga[2], Laura Foglia[4], Riccardo Mincigrucci[4], Alberto Simoncig[4], Carlo Spezzani[4], Claudio Masciovecchio[4], Tullio Scopigno[3,1*]

[1] *Graphene Labs, Istituto Italiano di Tecnologia, Via Morego 30 – 16163 Genova, Italy*

[2] *Center for Life Nano Science @Sapienza, Istituto Italiano di Tecnologia, Roma, I-00161, Italy*

[3] *Dipartimento di Fisica, Università di Roma "La Sapienza", I-00185, Roma, Italy*

[4] *Elettra-Sincrotrone Trieste S.p.C.A., SS 14-km 163.5, 34149 Basovizza, Trieste, Italy*

[5] *Dipartimento di Scienze Fisiche e Chimiche, Università degli Studi dell'Aquila, Via Vetoio, 67100 L'Aquila, Italy and*

[*] *Corresponding authors*

SUPPLEMENTARY INFORMATION

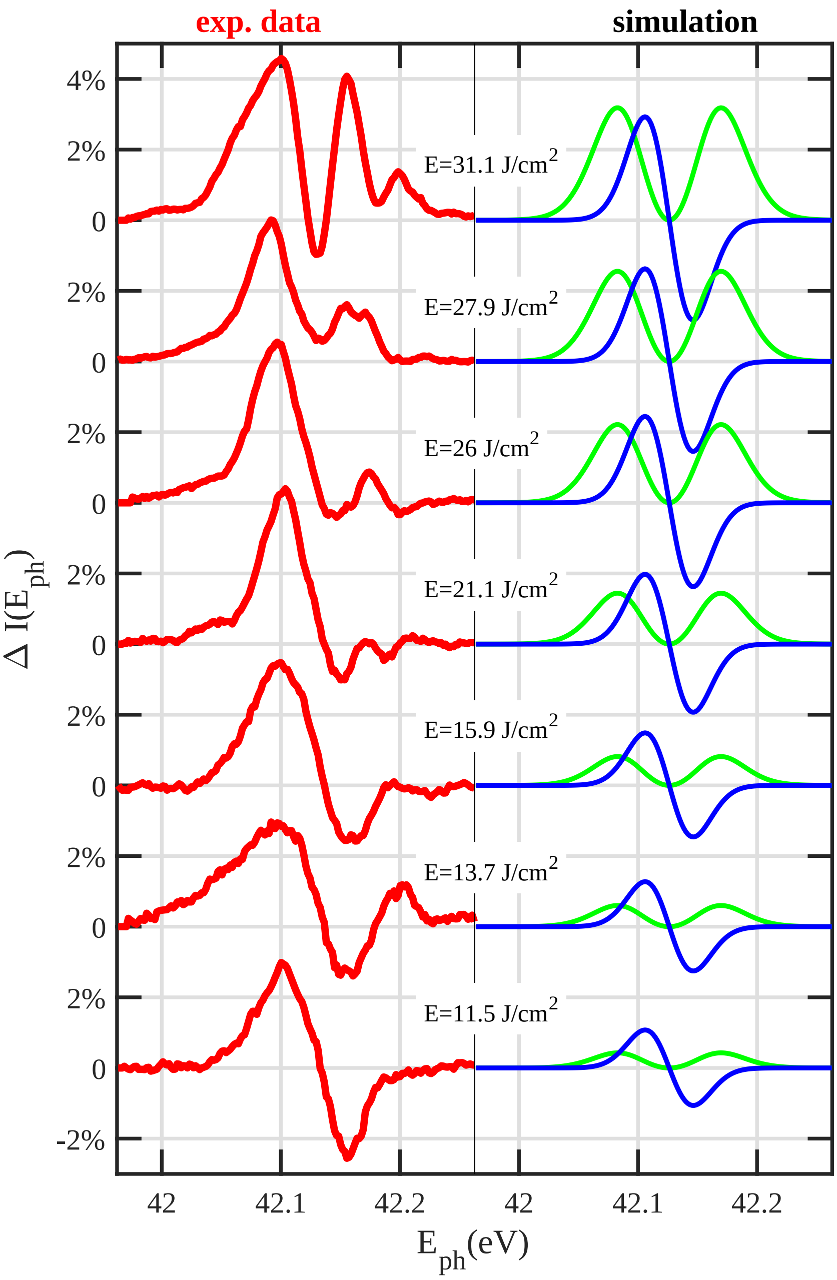

FIG. S1: **Theoretical simulations of experimental results by SPM and by DTRE.** The experimental data (red lines) reported for different FEL fluences are compared with the theoretical simulations evaluating separately only DTRE (blue lines) and SPM (green lines). The modification of the electromagnetic wave ($\Delta|\psi(L,\omega)|^2$) is calculated for $L = 140$ nm of Mg (see method section). Interestingly, both DTRE and SPM effects are not able to reproduce the experimental results. As shown in Fig. 2 of the manuscript, the simultaneous action of both the effects is required to reproduce the experimental data.

Single shot FEL spectra

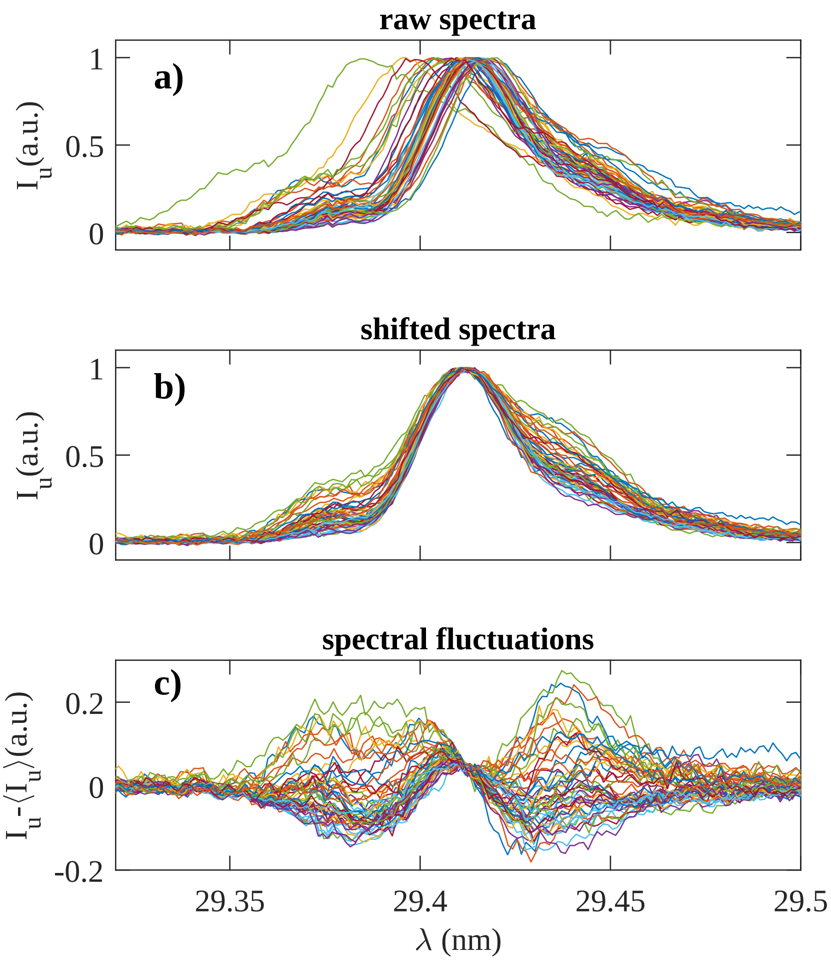

FIG. S2: **Single shot FEL spectra.** Raw FEL spectra collected by the upstream spectrometer (a) are compared with spectrally shifted spectra (b) and differential spectra (c).

Single pulse spectra measured upstream the sample are used as a reference to take into account for FEL fluctuations. Fig. S2 shows indeed that: (i) Single pulse spectra show non-negligible random offsets. (ii) Relative shifting single pulse spectra still does not result in a single profile, i.e. lineshape fluctuations as large as 10% are still present. Accordingly, the non-linear self-driven effect is extracted by evaluating the difference of a single downstream spectrum with respect to its upstream reference (shifted to maximize the spectral overlap). For what concerns the theoretical modelling of the FEL spectra, we have used a Fourier-transformed 59 fs Gaussian pulse. As shown in Figure S3, two stochastic spectral contributions are typically measured in the single shot spectral profiles. This latter is indeed

affected by satellite pulses with random temporal, spatial and spectral shift with respect to the main peak , as discussed in Fig. 12 of Ref[1]. In our simulation we have not considered the non-linear contribution arising from these weaker and unstable contributions.

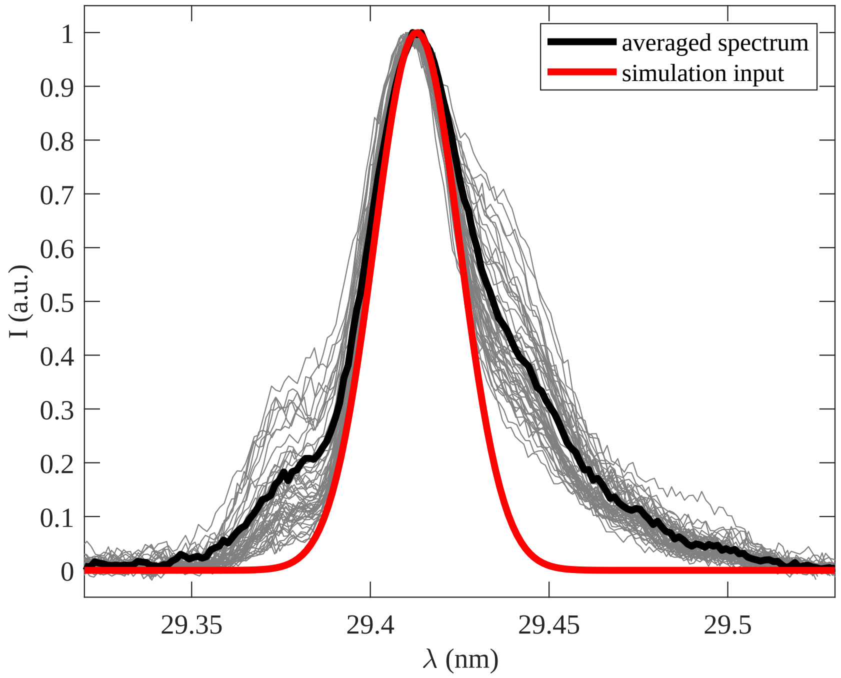

FIG. S3: **Comparison between experimental and simulations FEL spectral profiles.** The grey lines represent the FEL spectra (measured by the upstream spectrometer) for a specific power and the black line is their average. The red line is the spectral profile, obtained as the Fourier transform of a transform limited 59-fs pulse, used in the simulations.

1. Finetti, P. *et al.* Pulse duration of seeded free-electron lasers. *Phys. Rev. X* **7**, 021043 (2017).
